# Analysis of Mortality in Unvaccinated Patients with COVID-19 and Cardiovascular Risk

**DOI:** 10.3390/jcm11175004

**Published:** 2022-08-26

**Authors:** Kathie Sarzyńska, Filip Świątkowski, Jarosław Janc, Jan Zabierowski, Beata Jankowska-Polańska, Mariusz Chabowski

**Affiliations:** 1 Department of Nursing and Obstetrics, Faculty of Health Science, Wroclaw Medical University, 5 Bartla Street, 51-618 Wroclaw, Poland; 2 Department of Surgery, 4th Military Teaching Hospital, 50-981 Wroclaw, Poland; 3 Division of Anaesthesiologic and Surgical Nursing, Department of Nursing and Obstetrics, Faculty of Health Science, Wroclaw Medical University, 5 Bartla Street, 51-618 Wroclaw, Poland; 4 Department of Anesthesiology and Intensive Therapy, 4th Military Teaching Hospital, 50-981 Wroclaw, Poland; 5 Student Research Club No 180, Faculty of Medicine, Wroclaw Medical University, 50-556 Wroclaw, Poland; 6 Research and Innovation Center, 4th Military Teaching Hospital, 50-981 Wroclaw, Poland

**Keywords:** COVID-19, cardiovascular risk, laboratory test, mortality

## Abstract

COVID-19 is a contagious disease that has spread globally, killing millions of people around the world. In order to reduce the likelihood of in-hospital death due to COVID-19, it is reasonable to select a group of patients with a low probability of survival and to implement measures in advance to minimize the risk of death. One way to do this is to establish cut-off values for the most commonly performed blood laboratory tests, above or below which the likelihood of death increases significantly. The aim of the study was to determine the basic laboratory parameters among unvaccinated patients hospitalized for COVID-19 with concomitant cardiovascular disease, which are the predictors of in-hospital death. Out of 1234 patients, 446 people who met the specific inclusion criteria were enrolled in the study. The multivariate regression analysis has shown that the independent predictors of death are: troponin levels of at least 0.033 μg/L (OR = 2.04 [1.10; 3.79]), creatinine of at least 1.88 mg/dL (OR = 2.88 [1.57; 5.30]), D-dimers of at least 0.97 g/L (OR = 2.04 [1.02; 4.07]), and C-reactive protein minimum of 0.89 mg/L (OR = 2.28 [1.24; 4.18]).

## 1. Introduction

COVID-19 (coronavirus disease 2019) is an infectious disease of the respiratory system caused by infection with the SARS-CoV-2 virus. The first cases of the disease were recorded in December 2019 in the city of Wuhan, China. Since then, the number of COVID-19 cases has been growing dynamically and currently amounts to 267 million cases around the world confirmed by testing. So far, this disease has caused the death of as many as 5.3 million people [1].

The most common comorbidities with COVID-19 are cardiovascular diseases, which significantly increase the risk of a more serious course of COVID-19 and affect mortality. The vast majority of patients who have died due to COVID-19 had one or more comorbidities [2,3].

The relationship between the incidence of COVID-19 and cardiovascular diseases is confirmed by the increased incidence of complications in SARS-CoV-2 infection, such as myocardial infarction, arrhythmia, myocarditis, exacerbations of heart failure, Takotsubo cardiomyopathy, venous thromboembolism, and even cardiogenic shock. These complications are associated with a deteriorated prognosis and a higher risk of death [4,5]. Currently, numerous pathogenetic mechanisms are known to underline heart damage in COVID-19 infection. COVID-19 patients show an imbalance between oxygen supply and myocardial demand in response to severe hypoxemia, hypoperfusion and shock, as well as stress-induced cardiomyopathy. These factors may lead to acute cardiac injury, characterized by elevated troponin levels and cardiac dysfunction. Moreover, in the acute respiratory distress syndrome, the afterload of the right ventricle is influenced by positive pressure mechanical ventilation, reduced lung compliance and pulmonary vascular dysfunction, which leads to right ventricular dysfunction and acute cardiopulmonary syndrome [6].

Myocardial injury may also result from direct viral alteration of endothelial or myocardial cells, although detection of SARS-CoV-2 in these cells is rare [7]. Various virus entry receptors have been found, including the transmembrane protein ACE-2, in cardiomyocytes, endothelial cells, smooth muscle cells and fibroblasts, suggesting that the virus may, directly or indirectly, be responsible for cytopathic effects in the heart, even in people with apparently healthy hearts [8]. These cytopathic effects may worsen inflammation-induced endothelial dysfunction and the pro-thrombotic phenotype and may be responsible for microthrombosis in myocardial tissues. At the same time, the loss of ACE-2 and excessive activation of the renin–angiotensin–aldosterone system may contribute to endothelial dysfunction and multiorgan damage, including heart failure [6].

The analysis of clinical symptoms and laboratory tests in order to assess the impact of individual prognostic factors seems to be justified. In the group of patients with cardiovascular disease, this is important because it allows for early treatment correction, therapeutic interventions or faster hospitalization. In order to properly assess the patient’s condition and prognosis, readily available predictors of death due to SARS-CoV virus infection are searched for. There are many analyses of basic laboratory parameters in relation to the general population of COVID-19 patients [9,10]. Previous publications on similar topics have not been based on additional criteria for inclusion in the study in addition to a diagnosis of COVID-19. The authors of this study decided to admit patients with COVID-19 with concomitant cardiovascular disease or a risk factor for cardiovascular disease. Additionally, unvaccinated patients were selected for the study to assess the body’s response to the presence of a new pathogen, the SARS-CoV-2 virus. Patients were selected from the time period corresponding to the occurrence of the SARS-CoV-2 Delta mutation.

The aim of the study was to determine the relationship between the values of basic blood laboratory tests and in-hospital mortality in patients diagnosed with COVID-19. An attempt was also made to determine the cut-off points of the examined parameters, which would serve as alarm values, above or below which the risk of death increases significantly.

## 2. Materials and Methods

The study investigated the impact of selected laboratory blood parameters on in-hospital mortality. It was assumed that high levels of CRP, leukocytes, potassium, sodium, D-dimers, troponin and creatinine and low levels of hemoglobin and platelets should increase in-hospital mortality.

A hospital with an anonymous laboratory database of hospitalized patients was used for the research. An analysis of the medical data of 1234 patients in the period between November 2020 and February 2021 was performed.

**Figure 1:**
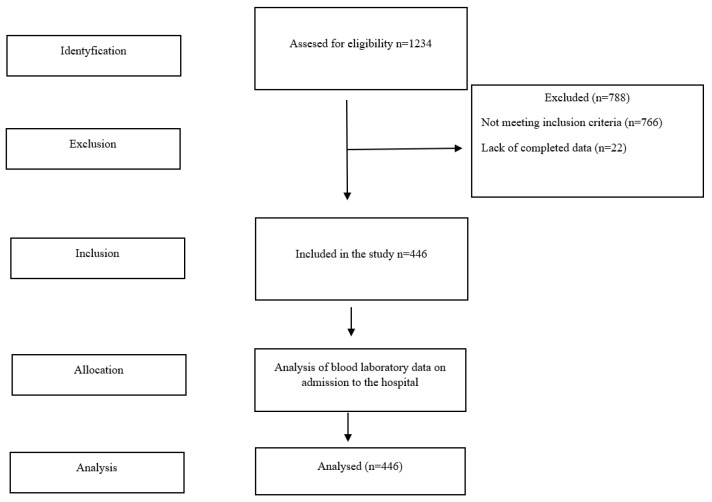
Study flow diagram.

The research included 446 patients who met the following inclusion criteria: positive PCR test for the presence of SARS-CoV-2 virus, the coexistence of at least one cardiovascular disease or cardiovascular disease development factor (diabetes, hypertension) and no vaccinations against COVID-19 prior to hospitalization. The process of recruiting patients for the study was carried out in several stages (Figure 1).

The study protocol was approved by the Bioethics Committee of the Wroclaw Medical University in Poland (permission no. KB-204/2021). The study was carried out in accordance with the guidelines of the Declaration of Helsinki and Good Clinical Practice.

Statistical methods. For quantitative variables, basic descriptive statistics were calculated (M—mean, SD—standard deviation, Me—median, Q1—lower quartile, Q3—upper quartile, Min—minimum value, Max—maximum value) and the compliance of their distributions with the theoretical normal distribution was checked using the Kolmogorov–Smirnov test and the Shapiro–Wilk test. Uni and multivariate logistic regression analyses were performed to establish statistically significant factors of the probability of death in the course of COVID-19 in the group of patients treated for pneumonia. The values of the regression coefficients b and their significance levels were estimated. ORs and their 95% confidence intervals were estimated. Kaplan–Meier charts were prepared to estimate the probability of survival of the studied group.

Continuous variables were dichotomized to establish independent predictors of death risk. The cut-off values were established based on the analysis of the ROC curves. A multidimensional logistic regression was performed. The values of the relative risk ratios (RR) were estimated. The fit of the logistic model to empirical data was verified using the Hosmer–Lemeshow test. A significance level of *p* < 0.05 was adopted for all statistical tests. Statistical analysis was performed using the STATISTICA v. 13.3 program (TIBCO, Software Inc., Palo Alto, CA, USA).

## 3. Results

Patients’ age ranged from 22 to 98 years, and the mean age (SD) was 72 (±13) years. Most of the respondents, n = 265 (59.4%), were men. In total, 224 patients (50.2%) were discharged home and the therapeutic process was completed. A total of 135 (30.3%) patients died during hospitalization. Every fifth person, n = 87 (19.5%), required further rehabilitation (Table 1).

In the study group, the most common comorbidities were cardiac arrhythmias (atrial fibrillation and other arrhythmias), which were diagnosed in 40% of patients (n = 170). The second most frequent disease was arterial hypertension in 29% of patients (n = 131), followed by diabetes in 17% of patients (n = 77). The median hospitalization was 12 days, and the mean number of comorbidities was two (Table 2).

The characteristics of the results of the laboratory parameters at the start of hospitalization are presented in Table 3.

### 3.1. Cut-Off Points of Laboratory Parameters

**Figure 2:**
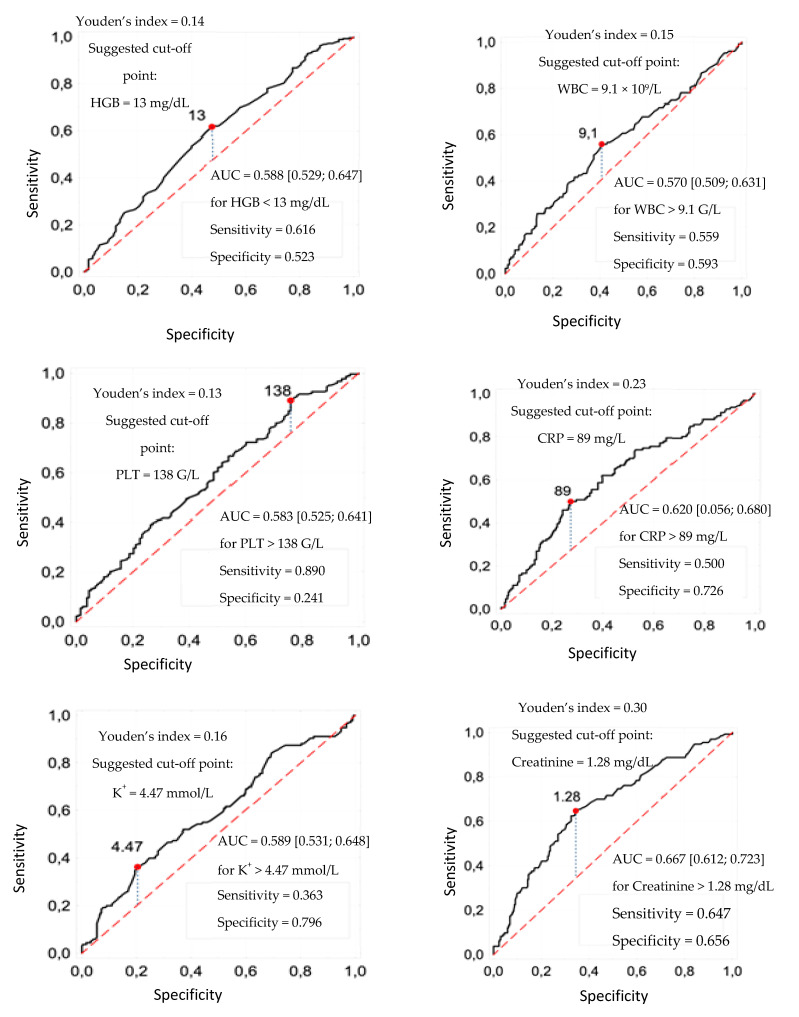

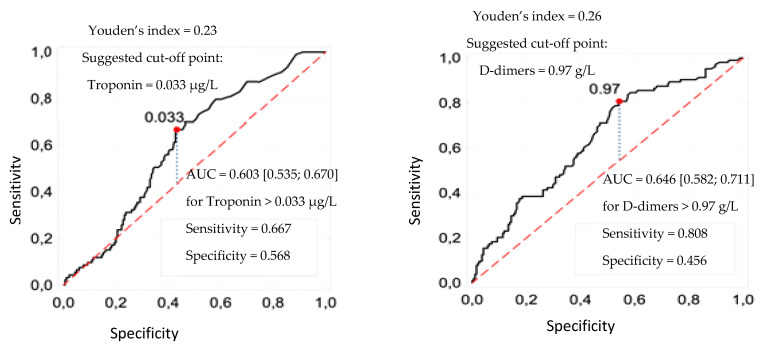
ROC curves for estimating in-hospital deaths based on blood counts and area under the curves (AUC) and their 95% confidence, sensitivity and specificity intervals for the threshold values proposed from the maximum Youden index.

The independent continuous variables, which are the results of blood counts, were transformed into dichotomous variables, establishing cut-off values based on the analysis of ROC curves. Cut-off points were determined for the following parameters: HGB, WBC, PLT, CRP, K^+^, creatinine, troponin and D-dimers, which were, respectively: 13 mg/dL, 9.1 g/L, 138 g/L, 89 g/L, 4.47 mmol/L, 1.28 mg/dL, 0.033 μg/mL and 0.97 g/L (Figure 2).

### 3.2. Predictors of Death

To determine which risk factors are independent predictors of death, a backward stepwise multivariate regression analysis (multiple regression) was performed. The model that allows estimation of the probability of a patient’s death (based on our study group) takes the following logit form:

logit P(death = 1|X) = −2.31 + 0.71 × (troponin ≥ 0.033) + 1.06 × (creatinine ≥ 1.28) +

+ 0.71 × (d-dimers ≥ 0.97) + 0.94 × (CRP ≥ 0.89).

Independent predictors of death were: troponin at least 0.033 μg/L, creatinine at least 1.28 mg/dL, D-dimer at least 0.97 g/L, and C-reactive protein at least 0.89 mg/L (Table 4).

### 3.3. Logistics Model Verification

**Figure 3:**
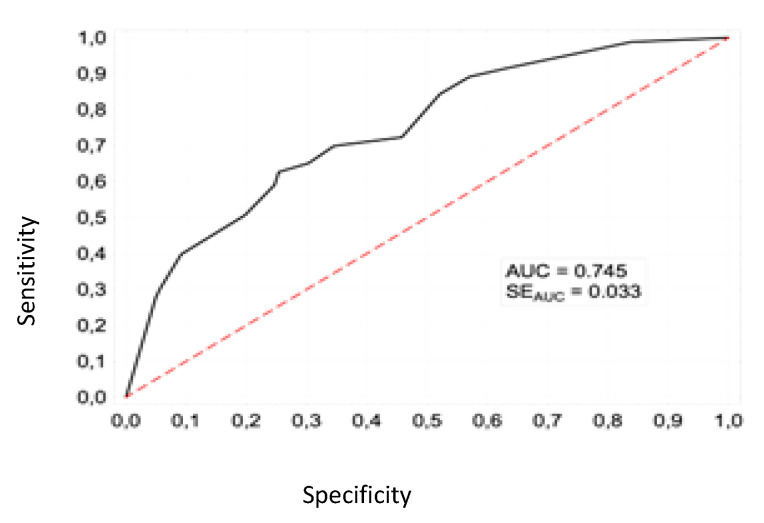
ROC curve for a model of the risk of death in patients with COVID-19 with cardiovascular disease.

The Hosmer–Lemeshow (HL) test was used to assess the adequacy of fit of the model to the empirical data. Its result: HL = 12.3 (*p* = 0.055) indicates that there is no reason to reject the null hypothesis and that there is no difference between the observed and predicted values of the dependent variable (death). This means that the model fits the data at an acceptable level. For the proposed model, the area under the ROC curve is AUC = 0.745 (Figure 3) which, according to the Kleinbaum and Klein (2010) classification, corresponds to sufficient discrimination.

### 3.4. The Survival Rate of Patients Depending on the Duration of Hospitalization

Kaplan–Meier survival curves were prepared to show the survival rate of patients depending on the duration of hospitalization. For this purpose, the patients were divided into two groups according to the cut-off points for individual values of laboratory parameters.

**Figure 4:**
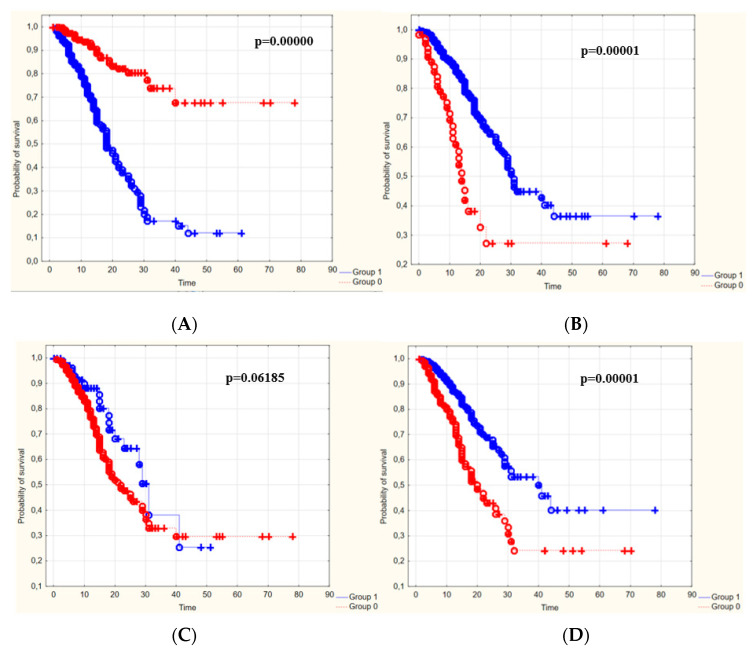

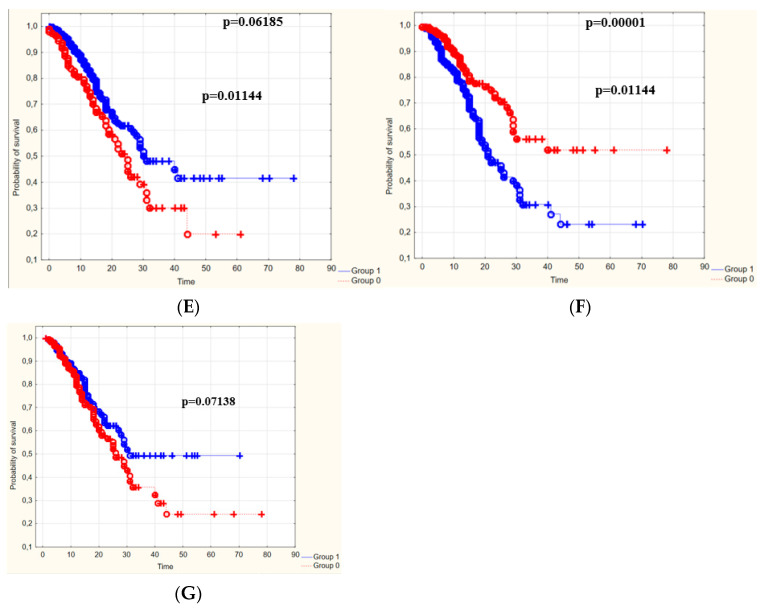
Kaplan–Meier survival curves for the cut-off points of individual laboratory parameters: (**A**)—WBC, (**B**)—PLT, (**C**,**D**)—dimers, (**D**)—CRP, (**E**)—K^+^, (**F**)—creatinine, (**G**)—HGB. Group zero—values below the cut-off point, group 1—values above the cut-off point.

The laboratory parameters important in estimating the probability of survival were the values of WBC, CRP, K^+^ and creatinine. Data analysis showed that patients with PLT values above the cut-off point and those with values below the cut-off point for WBC, CRP, K^+^ or creatinine during the entire hospitalization period were more likely to survive than the rest (Figure 4).

On the basis of Kaplan–Meier survival curves, a comparison of the survival of patients in the two groups (below and above the cut-off point) was performed for laboratory blood parameters such as WBC, PLT, D-dimer, K^+^, CRP and creatinine levels. The comparison was made on the 12th day of hospitalization, due to the fact that this was the median length of stay of the studied group of patients in the therapeutic center (Table 5).

The univariate analysis showed that the independent predictors of death in the study group were the values of the following laboratory parameters: WBC, PLT, CRP and K^+^. Moreover, significant differences in survival (Kaplan–Meier curves) in the two groups (below and above the cut-off point) were observed for laboratory parameters such as WBC, PLT, D-dimer, K^+^ and creatinine levels. The survival rate of patients in both groups was lower the longer the hospitalization time.

## 4. Discussion

The constantly growing number of new cases of COVID-19 disease and the reluctance of part of society to vaccinate against COVID-19 mean that the number of people requiring hospitalization due to a more severe course of the infection is considerable, especially in high-risk groups. Therefore, it is extremely important to find a method for the initial assessment of the patient’s risk of death upon admission to the hospital, and to look for prognostic factors of the disease. Identification of laboratory parameters that significantly correlate with the risk of in-hospital death may contribute to the selection of a group of patients with a significant risk of death and their early multispecialist in-hospital care.

In the age of universal cost calculation in healthcare, any action aimed at reducing treatment costs is desirable. Selecting high-risk patients and implementing early, intensive treatment will reduce the number of complications and the length of hospitalization. This will contribute to lowering the cost of the therapy. Importantly, all the laboratory parameters included in the study are cheap and easily available tests in every hospital. Their routine implementation during admission to hospital will not significantly increase hospitalization costs.

The present study investigated the relationship between the most frequently performed laboratory tests in previously unvaccinated COVID-19 patients with coexisting cardiovascular disease and in-hospital mortality. There are studies that prove that cardiovascular diseases are strongly correlated with a severe course of COVID-19 and with death [11].

A retrospective study of the nearly 73,000 COVID-19 cases in China found a death rate for the general population of 2.3%. However, for patients with hypertension, diabetes or cardiovascular disease, it was, respectively, 6%, 7.3% and 10.5% [12]. The influence of hypertension and other cardiovascular diseases on a severe course of COVID-19 and the mortality rate in the course of COVID-19 has been documented in studies [13,14]. A meta-analysis conducted by Almeida-Pititto et al. [11] showed a moderate association between COVID-19 mortality and the coexistence of diabetes mellitus and hypertension. This study also proved a strong relationship between the coexistence of cardiovascular diseases and mortality. The above findings may explain the high mortality rate in the study group of patients in our own work, which reached a high level of 30.3%.

Several studies have indicated a significant increase in WBC in non-survivors compared to survivors [15]. Such findings suggest a correlation between increased WBC and mortality. The increased number of leukocytes on admission to the hospital as a risk factor for death is consistent with the data in the currently available literature. There are also studies showing only correlations in the univariate model, thus failing to prove an independent correlation with mortality [9,10].

There are studies that prove that a reduced platelet count is an independent predictor of patient death, which is consistent with the results of the present study, in which the authors additionally set a platelet level cut-off of 138,000. Below this value, the risk of death increases, which is confirmed by the univariate analysis [16,17]. A study by Liao et al. [18] set a lower platelet level cut-off of 100,000. Such discrepancies may be caused, among other factors, by the fact that this study was completed during the first wave of the COVID-19 pandemic, when the predominant viral strain and the therapeutic approach were significantly different. The other reason may be the number of comorbidities and the median age of the research group. Only 48% of the subjects in the aforementioned study had any comorbidities, and the mean age of the patients was significantly lower than in our study group.

Several studies, as well as the current one, show that elevated CRP levels are an independent predictor of death [19,20,21]. CRP is a routinely used inflammatory biomarker produced and released by the liver in response to interleukin-6 stimulation. Increased CRP levels also appear to be an independent risk factor for the development of acute respiratory failure (ARDS) in COVID-19 patients, which may also significantly affect the risk of death [22]. This indicates a significantly increased risk of death and suggests a valuable prognostic value. In our study, the cut-off point for CRP was over 89 mg/L and was similar to the cut-off points in other studies, which were over 100 mg/L [23] and 81 mL/L [24]. A smaller value for the CRP cut-off point was obtained in the studies by Tahtasakal et al. [25] and Muinoz et al. [26], which were over 46 mg/L and over 4.5 mg/L, respectively. The differences in the obtained tests may be caused, among other factors, by the characteristics of our study group. In our own study, the mean age of the respondents and the percentage of in-hospital deaths were the highest.

The cut-off point for D-dimers in our own work was comparable to that established by Peiro et al. [23] and Muiños et al. [26], which, in these studies, was over 1,112 ng/mL and over 1,116 ng/mL, respectively. However, in the work of Tahtasakal et al. [25], this level was almost two times lower and amounted to over 574 ng/mL. However, in this study, the mean age of the patients was the lowest compared to the patients in all the above-mentioned studies. The younger the age, the lower the risk of dying from COVID-19 and the lower the likelihood of developing complications from COVID-19. Another study by Liao et al. [18] set a cut-off level to over 2 mg/L. This, however, may be connected to the number of people with comorbidities, which in the aforementioned study was about 48% compared to 100% in the present one.

In our study, the cut-off point value for troponin was established as over 33 ng/L. In other studies, the troponin cut-off point was lower and was over 7.8 ng/L [27], over 5.3 ng/L [25], over 4.56 [28] and over 21 ng/L [23]. All the above-mentioned studies confirm the relationship between the level of troponin and the risk of death. However, the values of the cut-off points differ from one another in the above-mentioned studies. Peiro et al. [23] obtained the most similar result to our own, where the study involved patients hospitalized in the emergency department. The possible explanation of such discrepancies may be the mean age, number of participants with comorbidities and mortality rate. Those variables differed depending on the study, but they were all consistently lower than in our study group. In addition, the patients with cardiovascular risk factors or cardiovascular disease may show elevated cardiac laboratory values compared to the patients without a similar disease burden.

The newly published Consensus Statement from the International COVID-19 Thrombosis Biomarkers Colloquium establishes elevated levels of CRP, D-dimers and reduced platelet count are significantly associated with in-hospital mortality, which supports our own data. Furthermore, all of the above-mentioned markers have a level of evidence of 1, which means that large, prospective studies including meta-analyses support those findings [29].

It should be emphasized that the studies conducted by others focused on a general group of patients with COVID-19. Other studies did not introduce any eligibility criteria for the study other than the diagnosis of COVID-19. Differences in results may be due to different responses of the organism vaccinated against COVID-19 compared to the unvaccinated. In addition, the presence of cardiovascular disease or a risk factor for cardiovascular disease (hypertension/diabetes), gender and older age may have a real impact on the results. The differences in the study groups may significantly affect the results due to the proven imbalance of the organism in the presence of many comorbidities. These disorders may have an impact on the pathogenesis and the course of COVID-19. For example, in hypertensive patients, both a generalized pro-inflammatory state and a weakened immune system can significantly worsen the prognosis of SARS-CoV-2 infection, thus affecting the values of laboratory parameters.

## 5. Conclusions

The values of the basic laboratory results of blood tests in COVID-19 patients with coexisting cardiovascular disease allowed the estimation of the risk of in-hospital death in this group of patients. Univariate analysis confirmed that all the laboratory parameters included in the study (HGB, PLT, WBC, CRP, D-dimers, troponin and creatinine) influenced the risk of death. On the other hand, the multivariate analysis showed that increased levels of WBC, CRP and K^+^ values and low PLT values were independent predictors of in-hospital death among cardiac patients or those with cardiovascular risk factors.

## 6. Practical Implications

The identification of the laboratory parameters that significantly affect in-hospital mortality in patients with COVID-19 and cardiovascular disease is particularly important from a clinical point of view. Early identification of people with abnormal laboratory test results allows for the intensification of treatment, which would reduce the risk of in-hospital death. Moreover, the determination of values (cut-off points) for laboratory parameters allows for an early initiation of therapy, which is often implemented only when the patient’s condition deteriorates and when the risk of death is already high.

## 7. Study Limitation

The study had some limitations. One of them was that the study focused on patients from one center. Another limitation was the rejection of some patients because they did not meet the inclusion criteria for the study. In addition, the study included a narrow group of people with COVID-19 who, apart from cardiovascular disease or cardiovascular disease development factors, also had other non-cardiac diseases that also influenced the clinical state of the patient and could be an additional determinant of in-hospital mortality, which was not included in this paper.

## Figures and Tables

**Table 1:** Characteristics of 446 participants.

Feature (Variable)	Statistics
Gender:	
Female, n (%)	181 (40.6)
Male, n (%)	265 (59.4)
Age (years of age):	
M ± SD	71.7 ± 13.2
Me [Q1; Q3]	72 [64; 82]
Min–Max	22–98
Hospitalization time (days)	
M ± SD	15.1 ± 11.6
Me [Q1; Q3]	12 [7; 20]
Min–Max	0–78
Discharge mode:	
Death, n (%)	135 (30.3)
End of the therapeutic process, n (%)	224 (50.2)
Referral for rehabilitation, n (%)	87 (19.5)

M—average; SD—standard deviation; Me—median (50%); Q1—lower quartile (25%); Q3—upper quartile (75%); Min—minimum value; Max—maximum value; n—group size; %—structure pointer.

**Table 2:** Characteristics of comorbidities in the study group.

Feature (Variable)	Number (%)
Comorbidities:	
Stroke	68 (15.2)
Pulmonary embolism	28 (6.3)
Atherosclerosis	20 (4.5)
Atrial fibrillation and other arrhythmias	170 (40)
Diabetes	77 (17)
Hypertension	131 (29)
Myocardial infarction	29 (6.5)
Ischemic heart disease	29 (6.5)
Embolism and arterial heart failure	11 (2.5)
Heart failure	68 (15.2)
Other	233 (52.2)
Number of comorbidities:	
M ± SD	2.0 ± 0.9
Me [Q1; Q3]	2 [1; 2]
Min–Max	0–5

**Table 3:** Characteristics of blood laboratory test results at the beginning of hospitalization.

Feature (Variable)	Statistics
HGB	
M ± SD	13.1 ± 2.3
Me [Q1; Q3]	13.2 [11.9; 14.5]
Min–Max	4.3–19.4
WBC	
M ± SD	9.8 ± 5.1
Me [Q1; Q3]	8.6 [6.3; 11.9]
Min–Max	0.9–33.4
PLT	
M ± SD	239 ± 109
Me [Q1; Q3]	221 [164; 288]
Min–Max	10–682
CRP	
M ± SD	75.0 ± 78.6
Me [Q1; Q3]	48.1 [11.9; 117.2]
Min–Max	0.1–475
Na^+^	
M ± SD	136 ± 5
Me [Q1; Q3]	137 [134; 140]
Min–Max	108–154
K^+^	
M ± SD	4.1 ± 0.7
Me [Q1; Q3]	4.0 [3.6; 4.5]
Min–Max	1.7–7.0
Creatinine	
M ± SD	1.53 ± 1.24
Me [Q1; Q3]	1.21 [0.96; 1.63]
Min–Max	0.44–15.13
Troponin	
M ± SD	1.77 ± 10.24
Me [Q1; Q3]	0.03 [0.01; 0.16]
Min–Max	0.0–100.84
D-dimers	
M ± SD	5.72 ± 14.74
Me [Q1; Q3]	1.29 [0.71; 3.24]
Min–Max	0.16–143.38

M—average; SD—standard deviation; Me—median (50%); Q1—lower quartile (25%); Q3—upper quartile (75%); Min—minimum value; Max—maximum value.

**Table 4:** Results of the logistic regression analysis of the risk of death.

Death Risk Factors	Univariate Analysis		Multivariate Analysis		
	*B*	*p*	*beta*	*p*	OR [95% CI]
HGB < 13 mg/dL	0.567	0.008			
WBC > 9.1 × 10^9^/L	0.612	0.004			
PLT < 138 × 10^9^/L	0.945	0.001			
CRP > 89 mg/L	0.943	<0.001	0.822	0.008	2.28 [1.24; 4.18]
K^+^ > 4.47 mmol/L	0.800	<0.001			
Creatinine > 1.28 mg/dL	1.249	<0.001	1.059	0.001	2.88 [1.57; 5.30]
Troponin > 33 ng/mL	0.965	<0.001	0.713	0.024	2.04 [1.10; 3.79]
D-dimers > 0.97 g/L	1.260	<0.001	0.712	0.044	2.04 [1.02; 4.07]

**Table 5:** Comparison of survival on day 12 of hospitalization for two groups of patients against the cut-off point for individual laboratory parameters.

Blood Laboratory Parameters [Cut-Off Point]	The Survival Rate of People with Values below vs. People with Values above the Cut-Off Point	The Survival Rate of People with Values above vs. People with Values below the Cut-Off Point
WBC [9.1 g/L]	32.8%	-
PLT [138 g/L]	-	43.3%
D-dimers [0.97 g/L]	10%	-
CRP [89 mg/L]	17.3%	-
K^+^ [4.47 mmol/L]	11.8%	-
Creatinine [1.28 mg/dL]	12.8%	-

## Data Availability

The data are available from the authors of the manuscript after contacting the corresponding author.
